# Challenges of *in vitro* modelling of liver fibrosis

**DOI:** 10.3389/fcell.2025.1567916

**Published:** 2025-04-30

**Authors:** Patricia Ros-Tarraga, Estela Villanueva-Badenas, Estela Sanchez-Gonzalez, Gloria Gallego-Ferrer, M. Teresa Donato, Laia Tolosa

**Affiliations:** ^1^ Experimental Hepatology Unit, Health Research Institute La Fe (IISLAFE), Valencia, Spain; ^2^ Faculty of Medicine and Dentistry, Department of Biochemistry and Molecular Biology, University of Valencia, Valencia, Spain; ^3^ Center for Biomaterials and Tissue Engineering (CBIT), Universitat Politècnica de València, Valencia, Spain; ^4^ Biomedical Research Networking Centre on Bioengineering, Biomaterials and Nanomedicine (CIBER-BBN), Carlos III Health Institute, Valencia, Spain; ^5^ Biomedical Research Networking Center in Hepatic and Digestive Diseases (CIBERehd), Carlos III Health Institute, Madrid, Spain

**Keywords:** liver fibrosis, extracellular matrix, *in vitro* systems, 3D models, therapies

## Abstract

Liver fibrosis has been proposed as the most important predictive indicator affecting prognosis of patients with chronic liver disease. It is defined by an abnormal accumulation of extracellular matrix components that results from necrotic and inflammatory processes and eventually impairs organ function. With no approved therapy, comprehensive cellular models directly derived from patient’s cells are necessary to understand the mechanisms behind fibrosis and the response to anti-fibrotic therapies. Primary human cells, human hepatic cell lines and human stem cells-derived hepatic stellate-like cells have been widely used for studying fibrosis pathogenesis. In this paper, we depict the cellular crosstalk and the role of extracellular matrix during fibrosis pathogenesis and summarize different *in vitro* models from simple monolayers to multicellular 3D cultures used to gain deeper mechanistic understanding of the disease and the therapeutic response, discussing their major advantages and disadvantages for liver fibrosis modelling.

## 1 Introduction

The most prevalent chronic liver disease in the world, metabolic-dysfunction associated liver disease (MASLD), affects 32% of adults (Riazi et al., 2022), and is characterized by increased fat accumulation. Its overall prevalence is significantly higher in men than in women and has increased over the years (Riazi et al., 2022). There are two distinct histological types: a) simple steatosis, which is defined as the presence of fat droplets, primarily triglycerides, in the liver without hepatocellular necrosis and without, or minimal, inflammation, and b) metabolic dysfunction-associated steatohepatitis (MASH), which is characterized by fat accumulation, liver inflammation, and hepatocyte ballooning with or without fibrosis (EASL EASD EASO, 2016). The advancement of MASH can lead to cirrhosis and, eventually, hepatocellular carcinoma (EASL EASD EASO, 2016).

Fibrosis has been suggested as the most significant predictive factor influencing prognosis and it has been closely linked to liver transplantation and liver-related death in patients with MASLD (Angulo et al., 2015). Major regulatory authorities such as the Food and Drug Administration and European Medicines Agency are ordering trials in advanced MASH (Schwabe et al., 2020).

Fibrosis is the result of excessive production of extracellular matrix (ECM) that is not sufficiently counterbalanced by degradation, thus resulting in its accumulation (Schwabe et al., 2020). The activation of hepatic stellate cells (HSCs) is an important event in hepatic fibrosis. After liver injury, quiescent HSCs are activated and differentiate into myofibroblasts. After excessive proliferation of myofibroblasts, a large amount of ECM such as collagen is synthesized, accompanied by increased matrix cross-linking and insufficient ECM degradation, eventually leading to liver fibrosis (Chen et al., 2019). Liver failure after long standing cirrhosis is caused by loss of several critical functions of hepatocytes such as synthesis and secretion of plasma proteins, storage of biomolecules and micronutrients, regulation of glucose homeostasis, metabolism of drugs and blood detoxification. However, hepatocyte loss of function is caused not only by the direct viral or toxic insult to hepatocytes themselves but is also highly exacerbated by the disruption of the cellular interaction network wherein hepatocytes reside (Marrone et al., 2016; van Riet et al., 2024). Interactions with other non-parenchymal cells (NPC) in this network include HSCs, liver sinusoidal endothelial cells (LSECs) and liver macrophages (including both Kupffer Cells (KCs), and peripheral blood macrophages). Therefore, comprehensive models including numerous cell types would be necessary to understand the mechanisms behind liver fibrosis (Kumar et al., 2021).

Lifestyle modification is the primary intervention for the treatment of fibrosis; thus, research efforts are focused on developing safe and effective treatments to improve clinical management of the disease (Diehl and Day, 2017). The control of the mechanisms involved in the initiation and progression of the disease such as chronic inflammation, insulin resistance and fibrogenesis, is the primary focus of research to find new therapeutic targets for liver fibrosis (Diehl and Day, 2017; Thiagarajan and Aithal, 2019). However, although some anti-fibrotic candidates have shown robust effects in animal models, the number of clinical trials is limited, and no approved therapy exists for liver fibrosis. Similar to the multicellular network involved in fibrogenesis, fibrosis resolution involves multiple cell types that should be considered when designing and testing new therapies. Moreover, it is imperative that future studies standardize clinical endpoints and fibrosis measurements for a better understanding of drug efficacy.

Detailed understanding of liver diseases is limited by the lack of appropriate disease models that reveal the molecular mechanisms implicated; thus, the development of suitable and reproducible liver tissue models is fundamental for regenerative medicine, drug screening and disease modeling. The current *in vitro* liver disease test systems comprise simple cell-based models to more complex three-dimensional (3D) organoids. Primary human HSCs are the gold standard for modeling liver fibrosis, although their use is hindered by the increasing shortage of suitable donor livers for their isolation as well as by the insufficient functional quality; thus, new cell-based models are being developed. In this sense, considering that liver fibrosis is a complex and multifaceted pathological process involving the interactions among various cell types, signaling pathways, and biomechanical changes, new 3D and multicellular models offer significant advantages for modelling liver fibrosis *in vitro*. This review summarizes recent advances in cell-based models of liver fibrosis and aims to overview the relevant mechanisms and challenges in the development of anti-fibrotic treatments.

## 2 Liver extracellular matrix

### 2.1 Composition and role of ECM in the liver

The ECM, while a minor constituent of the liver, plays a critical role in providing structural support and regulating cell and tissue homeostasis (Martinez-Hernandez and Amenta, 1993; Ortiz et al., 2021). The ECM forms a fibrous network that facilitates cell adhesion, provides space for cell growth and migration, and serves as a reservoir for signaling molecules (Baiocchini et al., 2016). This interaction is mediated by cell surface receptors, like integrins, that can signal through the cell membrane in either direction and regulate cell adhesion, migration, proliferation, apoptosis, survival or differentiation (Mezu-Ndubuisi and Maheshwari, 2021; Ortiz et al., 2021).

The liver matrisome consists of more than 150 different ECM proteins and ECM-associated proteins, such as collagens, elastins, fibronectins, and laminins, responsible for the cellular phenotype and function (Ortiz et al., 2021). It is also composed by ECM regulators and modifiers, like matrix metalloproteinases (MMPs) and secreted factors that bind to the ECM, like transforming growth factor-β (TGF-β) and other cytokines (Arteel and Naba, 2020). ECM can be divided into two structurally distinct types: an epithelial/endothelial basement membrane and interstitial matrix. The most abundant components of the basement membrane are laminins, nidogen/entactin, non-fibrillar collagens, like collagen type IV, VIII and X, and heparan sulfate proteoglycans. Instead, the interstitial matrix is mainly composed by elastin, fibronectin, tenascin, and the fibrillar collagens type I, II, III and V (Karsdal et al., 2013; Karsdal et al., 2017; Lonsmann et al., 2023). Furthermore, a unique ECM separates the sinusoidal endothelium from the epithelial hepatocytes, the basement membrane-like matrix, which contains basement membrane constituents and non-basement membrane components (like type I collagen and fibronectin). This unique composition is essential for hepatocytes’ viability and function, and changes in it are associated with hepatic failure (Zeisberg et al., 2006).

### 2.2 ECM in liver fibrosis

Regardless of the cause, hepatic fibrosis is characterized by an increase in ECM constituents caused by an unbalanced chronic wound-healing process, which affects its structure and biophysical properties. As a consequence, hepatic scar appears and tissue fibrosis develops (Ortiz et al., 2021; Walraven and Hinz, 2018).

A fibrous scaffold is usually formed as a result of pathological ECM remodeling, increasing local tissue stiffness and affecting the behavior of surrounding cells through mechanical forces (Zhang et al., 2024). Clinically, patients with liver fibrosis who have increased hepatic stiffness as a result of excessively deposited highly insoluble matrisome are at risk for decompensation, liver malignancy, and even mortality (Zhang et al., 2024).

Increased *de novo* collagen production is the main characteristic of fibrogenesis (Arteel and Naba, 2020). Consequently, variations in collagen synthesis/deposition biomarkers could be used to forecast the severity of liver illness. For instance, it has been determined that the Type III collagen precursor has areas under the receiver operating characteristics curve (AUROC) values for predicting the severity of liver disease that are better than imaging and/or scoring methods (Arteel and Naba, 2020). Moreover, ECM remodeling in the matrisome components, their covalent intra- and intermolecular crosslinking, and the alteration of the chemical and mechanical microenvironment of the ECM are common features of liver fibrogenesis (Chen et al., 2023). Certain proteins, such as elastin, aggregate and stabilize in fibrotic livers, increasing density and stiffness, preventing fibrinolysis and changing liver homeostasis (Chen et al., 2023; Karsdal et al., 2017).

On the other hand, using proteomics Daneshgar and cols (2020) revealed a significant fraction of 70 distinct matrisome proteins that are widely expressed in both healthy and fibrotic and cirrhotic liver scaffolds. Additionally, the expression of 59 distinct matrisome proteins varied between livers in various stages of fibrosis and healthy livers. Some of these matrisome proteins’ expression may hold promise for the creation of tissue or serological biomarkers and possible liver fibrosis treatment targets. Finally, compared to their healthy counterparts, 19 different matrisome proteins were completely reduced in all phases of fibrosis. Among these proteins, hemopexin plays a crucial role in metabolism and inflammation (Daneshgar et al., 2020).

The progression of liver fibrosis depends, among other things, on the cross-links of the different collagens in the basement membrane and the interstitial matrix (Brown et al., 2006). Liver fibrosis also increases the type IV collagen, laminin and nidogen (Hudson et al., 2003). Changes in the quantity and the quality of collagen crosslinking, which produces an increase in their stiffness, are produced by different enzymes, like lysyl oxidases (LOX) and transglutaminases (TG), or by non-enzymatic glycation (Hynes and Naba, 2012). Also, collagens and other ECM proteins have proteolytic cleavage due to MMP, A disintegrin and metalloproteinases (ADAM) and ADAM with thrombospondin motives proteases, as well as other proteolytic enzymes like elastases, cathepsins, and different serine esterase proteases (Kisseleva and Brenner, 2021; Lu et al., 2011). This formation of different collagens during liver fibrosis induces myofibroblast activation, the main producers of ECM. These cells, together with hepatocytes and macrophages, regulate hepatic fibrogenesis and its progression. Another important signaling peptide derived from collagen XVIII, located in the basement membrane, is endostatin. This collagen is produced by hepatocytes and is associated with advanced stages of liver fibrosis (Ding et al., 2014; Kisseleva and Brenner, 2021).

## 3 Mechanisms of liver fibrosis

### 3.1 The key role of hepatic stellate cells in liver fibrosis

Liver fibrosis is a complex pathological process that virtually involves all cell types present in the liver, although the key role of HSCs is widely recognized. Activated HSCs are the main responsible of excessive accumulation of ECM in injured liver tissue, although other cell populations such as bone-marrow derived myofibroblasts or portal fibroblasts may also contribute to ECM deposition (Bataller and Brenner, 2005; van Grunsven, 2017).

HSCs are mesenchymal type cells located at the space of Disse, the perisinusoidal space of the liver between hepatocytes and LSECs. Under physiological conditions, they are in a quiescent status showing a characteristic dendritic morphology with cytoplasmatic extensions that promote their interactions with the surrounding cells (Sato et al., 2003). Quiescent HSCs contain large lipid droplets containing vitamin A and have important roles in the regulation of retinoid homeostasis, the physiological synthesis of ECM components and the production of MMPs responsible of ECM remodeling (Sato et al., 2003).

As a result of liver injury, quiescent HSCs undergo an activation or transdifferentiation process acquiring a myofibroblast-like phenotype. Hallmarks of HSCs activation are the loss of lipid droplets and the presence of new phenotypic features such as the expression of α-smooth muscle actin (αSMA) and other myofibroblastic markers (Higashi et al., 2017). In this activation process two phases, termed initiation and perpetuation, are considered (Friedman, 2000). During initiation, HSCs undergo early gene expression changes rendering cells more responsive to extracellular stimuli. Perpetuation step involves several cellular and phenotypic changes (proliferation, migration, contractility, ECM secretion, retinoid loss, cytokine release) that amplify HSC activation and accelerates ECM accumulation. Both initiation and progression are multifactorial processes regulated by a variety of cell-cell and cell-biomatrix interactions, and by multiple chemical mediators and signaling pathways (Bataller and Brenner, 2005; Higashi et al., 2017). Once activated, HSCs exhibit profibrinogenic and proinflammatory characteristics with production of fibrillary collagens (type I and III), tissue inhibitors of MMPs (TIMPs), cytokines and growth factors (Wang et al., 2022).

In advanced disease stages, the liver matrix undergoes marked increases in the content of collagen and other ECM components, such as laminin, elastin, fibronectin and hyaluronic acid (Matsuda and Seki, 2020). ECM accumulation is the result of dysregulated synthesis and remodeling functions in HSC. MMPs, the main collagen degrading enzymes, are downregulated in activated HSCs while TIMP-1 and -2, specific inhibitors of MMP enzymes, are overexpressed, which results in a blockage of ECM degradation and fibrosis progression. Other enzymes, such as LOX and TG, are also upregulated during liver fibrosis, promoting protein cross-linking and/or stabilization of ECM proteins (e.g., collagen I or fibronectin), increasing their resistance to proteolytic degradation (Ortiz et al., 2021). Therefore, net outcomes of sustained HSC activation are increased ECM deposition and changes in ECM composition due to the formation of pathological fibrillary collagen forms.

### 3.2 Role of cell-cell and cell-matrix interactions in HSC activation and liver fibrosis

**FIGURE 1:**
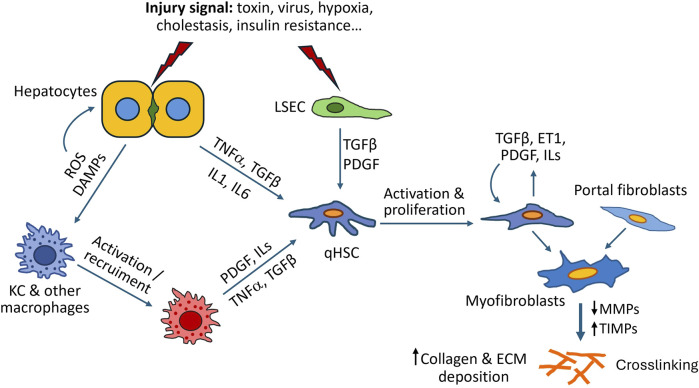
Mechanisms of liver fibrosis. As a result of liver injury, damaged hepatocytes release inflammatory cytokines, reactive oxygen species (ROS), damage-associated molecular patterns (DAMPs) and other soluble factors, that directly, or indirectly with the contribution of other cells such as Kupffer cells (KC) or liver sinusoidal endothelial cells (LSEC), can trigger activation of hepatic stellate cells (HSC) into myofibroblast-like cells. Activated HSCs synthesize large amounts of ECM components and secrete additional cytokines that perpetuate their activated state. Excessive ECM accumulation and liver fibrosis are the result of dysregulated ECM synthesis and remodelling due to downregulation of MMPs (matrix metalloproteinases) and increased production of TIMPs (tissue inhibitors of MMPs).

Hepatocyte injury is a key initial event in liver fibrosis. In the context of diverse chronic liver diseases, damaged hepatocytes release reactive oxygen species (ROS), damage-associated molecular patterns (DAMPs), or soluble factors, that directly, or indirectly with the contribution of diverse NPCs, can trigger HSC activation (Bataller and Brenner, 2005; Lee and Friedman, 2011; Liedtke et al., 2021) (Figure 1). ROS and other damage signals from hepatocytes activate KCs and monocyte-derived macrophages leading to further release of proinflammatory mediators such as transforming growth factor β (TGF-β), platelet-derived growth factor (PDGF), tumor necrosis factor α (TNFα), interleukin (IL)-1β and IL-6 (Bataller and Brenner, 2005; Liedtke et al., 2021; Ying et al., 2017). In response to these stimuli quiescent HSCs become activated through specific receptors and signaling pathways, leading to increased ECM deposition. Other cell populations, such as natural killers, lymphocytes, proliferating bile duct epithelial cells, cholangiocytes and LSECs can also drive fibrosis progression (Higashi et al., 2017; Zhang et al., 2022). Normal LSECs degrade ECM components, playing an important homeostatic function. When damaged, LSECs lose their typical fenestrae and form a basement membrane. These structural alterations are accompanied with the loss of ECM degradative capacity and release of soluble factors, such as TGF-β or PDGF, that activate neighboring HSCs in a paracrine manner (Ni et al., 2017). In addition to paracrine activation of HSCs triggered by other cell types, activated HSCs secreted chemokines (e.g., TGF-β, PDGF and endothelin-1) that contribute to perpetuating their activated state in an autocrine loop (Friedman, 2000; Lee and Friedman, 2011).

Progressive changes in the composition and mechanical properties of ECM serve as additional stimuli for HSC activation. Fibrotic tissue is characterized by an increase of ECM stiffness, due to an excessive deposition and crosslinking of extracellular proteins, which affects cell behavior. Fibrillar collagens type I and III can directly interact with surface adhesion receptors, integrins, and mediate profibrotic pathways and intracellular response (Hudson et al., 2017). Integrin-signaling plays an essential role in the cleavage and activation of latent TGF-β, considered a major fibrogenic chemokine in the liver (Fan et al., 2019; Ortiz et al., 2021).

The most prominent phenotypic changes associated with the transformation of HSCs into myofibroblast-like cells are synthesis of type I and III collagen, expression of αSMA, production of TGF-β, augmented expression of TGF-β receptors and release of TIMPs 1 and 2. In addition to αSMA, vimentin and desmin are phenotypic features of activated HSCs commonly used as histological/immunological markers of liver fibrosis. All these phenotypic changes are accompanied by alterations in the expression of several genes (e.g., *COL1A1* and *2, COL3A1, ACTA2, MMP1, LOX*) and are strictly regulated by signaling pathways, such as TGF-β, PDGF or Toll-like receptor pathways (widely reviewed in (Higashi et al., 2017)).

Discontinuation of the stimulus of liver damage and remission of the tissue injury can lead to resolution of fibrosis, which involves both elimination of activated HSCs (by apoptosis, senescence or regression to quiescent phenotype), and degradation of fibrotic ECM with active participation of macrophages and LSECs (Matsuda and Seki, 2020; Ni et al., 2017).

Despite numerous studies focused on liver fibrosis, currently, its pathogenesis remains to be insufficiently elucidated. Further research efforts are needed to increase the understanding of the cellular and molecular mechanisms driving the initiation, progression and regression of fibrosis to contribute to the identification of new diagnostic markers and therapeutic agents with clinical application in the management of patients with chronic liver diseases.

## 4 Cell culture models for liver fibrosis study

Liver fibrosis is caused by a variety of cell types, and animal models have been traditionally used for understanding the disease’s mechanism. However, the interpretation of *in vivo* models is sometimes complex and difficult to extrapolate to a clinical context. On the other hand, human cell-based models are easier to use and helpful in comprehending the molecular processes behind HSC activation (Lee and Seki, 2023). Table 1 summarizes the main *in vitro* models used in the study of liver fibrosis.

**TABLE 1:** Major cellular models for studying liver fibrosis.

*In vitro* model	Highlights	Advantages	Disadvantages
Primary HSCs	➢ Isolated from human tissue ➢ Both HSC and myofibroblasts can be isolated from healthy and injured livers, respectively ➢ Response to profibrotic stimuli such as TGF-β or IL17 A	➢ Closer to the *in vivo* situation ➢ Maintenance of key functions, i.e., storage of vitamin A in lipid droplets	➢ Technical difficulties in the isolation process. Low yield ➢ Reduced lifespan ➢ Contamination with other NPC ➢ Activated when cultured in plastic plates ➢ Shortage of tissue for isolation
HSC lines	➢ Immortalized from primary HSCs ➢ Response to profibrotic stimuli such as TGF-β, IL-17 or PDGF	➢ Availability ➢ Easy handling	➢ Loss of some functions (i.e., lipid droplets) and typical morphology ➢ Basal activation (expression of profibrotic genes)
iPSC-derived HSC	➢ Differentiation with a multi-step protocol ➢ Response to profibrotic stimuli such as TGF-β or PDGF.	➢ Derived from patients ➢ Reflect variability in the population ➢ Disease modelling	➢ Immature phenotype ➢ Variability in the efficiency of differentiation
Co-cultures	➢ Culture of different hepatic cell types ➢ Response to profibrotic stimuli such as TGF-β	➢ Closer to the *in vivo* situation allow to study the contribution of different cell types ➢ Allow the analysis of cell-cell interactions	➢ Normally, are simply based on hepatocytes and HSC ➢ Reduced functionality if cell lines are used
Liver organoids	➢ Derived from human tissue or iPSC ➢ Response to profibrotic stimuli such as TGF-β	➢ 3D organization ➢ Disease modelling ➢ Allow the analysis of cellular interactions	➢ Shortage of tissue for isolation ➢ Restricted maturity
Precision-cut Liver slices	➢ Liver explants ➢ Response to profibrotic stimuli such as TGF-β	➢ Closer to the *in vivo* situation ➢ All cell populations are present	➢ Limited lifespan

3D, three-dimensional; HSC, hepatic stellate cells; IL, interleukin; iPSC, induced pluripotent stem cells; NPC, non-parenchymal cells; PDGF, platelet-derived growth factor; TGF-β, transforming growth factor-β.

### 4.1 Primary hepatic stellate cells

Primary HSCs are the gold standard for modeling liver fibrosis. *In vitro*, in a quiescent state, these cells keep key functions such as the storage of vitamin A in lipid droplets (Zhai et al., 2019) and the expression of genes involved in maintaining ECM stability (Lua et al., 2016).

*In vitro* models of liver fibrosis frequently involve exposing HSCs to profibrotic molecules and cytokines. Stimulation with PDGF or TGF-β plays a key role in driving HSC proliferation, migration, and activation of MMPs, processes that are essential for hepatic matrix remodeling (El Taghdouini et al., 2015). Moreover, TGF-β suppresses the expression of genes associated with the quiescent state, such as *PPARγ*, while simultaneously upregulating genes linked to ECM remodeling, including *ACTA2*, *COL1A1*, *PDGFR-β*, *VIM*, *TIMP-1*, and *LOX*. These molecular changes are accompanied by increased production of type I collagen and α-SMA, hallmarks of the fibrotic response (Lin et al., 2006; Marti-Rodrigo et al., 2020).

Furthermore, IL-17 A amplifies the HSC response to TGF-β by upregulating the expression of its receptor on the cell surface *via* a JNK-dependent pathway. This leads to increased production of type I collagen, α-SMA, and TIMP-1, ultimately exacerbating fibrosis (Fabre et al., 2014).

The use of primary HSCs has shown promise for modeling *in vitro* liver fibrosis, but it comes with certain limitations. Firstly, the yield of isolated HSCs is low, and their proliferation rate in the quiescent state is minimal (Meurer et al., 2023). Additionally, the quiescent phenotype is difficult to maintain *in vitro*, as the cells begin to activate spontaneously after 4–5 days in culture. During this activation, HSCs develop pseudopodial morphology, lose their lipid droplet stores, and exhibit increased expression of *COL1A1*, *α-SMA*, *FN1*, and *COL2A1* (Chang et al., 2014; Sekiya et al., 2011).

### 4.2 Hepatic stellate cell lines

Immortalized cell lines have been developed to address the limitations of primary HSCs. These immortalized HSC lines offer several advantages, including unlimited proliferation, lower costs, and a more consistent phenotype. Among the most used cell lines for liver fibrosis research are the GRX line, derived from mouse liver (Borojevic et al., 1985); the HSC-T6 line, derived from rat stellate cells (Vogel et al., 2000); and human lines such as LX-2, LX-1, and LI90 (Murakami et al., 1995; Xu et al., 2005).

Like primary HSCs, HSC cell lines respond to profibrotic stimuli such as TGF-β and PDGF. Exposure to TGF-β in LX-2 cells increases the expression of myofibroblast markers, such as MMP-2, TIMP-1, α-SMA, endothelin-1, PDGF-BB, type IV collagen α1, and type I collagen α1, both at the mRNA and protein levels. Additionally, TGF-β suppresses the expression of matrix-degrading proteins like MMP-1 and MMP-3, while also upregulating proinflammatory cytokines and chemokines, including IL-1β, TNF-α, CXCL1, and CCL2 (Robert et al., 2016). Similarly, exposure to IL-1β mimics the effects of TGF-β, boosting the production of vimentin, fibronectin, and α-SMA, as well as enhancing both the expression and enzymatic activity of MMP-2 (Masola et al., 2019).

A key component in the profibrotic mechanism triggered by TGF-β in LX-2 cells is the AGAP2 protein. AGAP2 regulates LX-2 activation by modulating critical effects induced by TGF-β1, such as proliferation, migration, and the expression of profibrotic genes. Furthermore, AGAP2 promotes the increase of collagen I production in LX-2 cells through FAK activation and influences the trafficking of the TGF-β type II receptor, which sustains signaling and accelerates fibrosis progression (Navarro-Corcuera et al., 2019).

In an *in vivo* profibrotic environment, various liver cells contribute to the production of proinflammatory cytokines like IL-17. *In vitro*, both IL-17 A and IL-17 F enhance the profibrotic effects of TGF-β through SMAD2/3 activation, increasing the expression of *TGFBRII* in LX-2 cells. Blocking IL-17 R A reduces the surface expression of TGFBRII on LX-2 cells and decreases the expression of profibrotic markers, such as *COL1A2* and *TIMP1*, demonstrating the synergistic profibrotic interaction between these two stimuli (Matsuda et al., 2024).

Despite their advantages over primary HSCs, these cell lines have certain limitations. They have lost the typical morphology of HSCs and some of their functions. Furthermore, they more closely resemble myofibroblasts, lacking lipid droplets, and are more activated than primary HSCs, as they express profibrotic genes at levels comparable to those found in activated HSCs (Herrmann et al., 2007).

### 4.3 Co-cultures

HSCs are the main drivers of liver fibrosis, but the development and progression of this pathology relies on interactions among various liver cell types. An ideal *in vitro* model would have functional hepatocytes that can be harmed by a chemical and HSCs that can activate towards a myofibroblast phenotype. Repeated hepatocyte cell death (or damage) triggers a reaction that aims to restore the liver’s structure and function. This response includes macrophages clearing the dead hepatocytes, HSCs producing ECM to stabilize the hepatic architecture, and hepatocyte regeneration to replace the damaged cells. Moreover, although it is clear that hepatocyte injury dead can trigger fibrogenesis, it can also trigger stress reactions in a number of hepatic cell types, which in turn trigger fibrogenesis (van Grunsven, 2017). *In vitro* cultures of hepatocytes and HSC have been widely used for modelling liver fibrosis, being primary human hepatocytes (PHH) the most common source of hepatocytes, although human hepatocyte cell lines or stem cell-derived hepatocytes are also commonly used (van Grunsven, 2017). The most crucial limiting aspect of using PHH is that once the cells are plated, they cannot be maintained in their original form quickly lose their differentiated phenotype and do not proliferate.

On the other hand, hepatocytes derived from stem cells are not fully mature. Profibrotic substances released by damaged hepatic cells can activate HSCs. Replicating these interactions *in vitro* is essential to understanding the initiation and progression of liver fibrosis.

Acetaminophen (APAP) is a widely used drug for pain relief, and it is metabolized by the enzymes CYP2E1 and CYP3A4 into NAPQI, a toxic and profibrotic compound. The inflammatory component plays a critical role in its toxicity. When APAP is combined with cytokines such as TNF-α, IL-1β, IFN-γ, and IFN-α in a model of primary HSCs co-cultured with the HepaRG line (hepatocarcinoma cells that retain many characteristics of primary human hepatocytes), it produces a more pronounced increase in the expression of *COL1A1, COL3A1*, and *LOXL2* compared to APAP alone, suggesting a synergistic profibrotic effect (Leite et al., 2016).

Similarly, other fibrotic compounds such as methotrexate and allyl alcohol after repeated exposure in spheroids of primary HSCs and HepaRG cells increases *COL1A1* and *LOXL2* expression and pro-collagen type I production, exceeding levels seen in HSC monocultures (Leite et al., 2016).

Co-culture systems with greater complexity allow the analysis of cellular interactions in liver fibrosis development. For example, co-cultures of HepaRG, LX-2, and human umbilical vein endothelial cells (HUVEC) in agarose microwells have shown that TGF-β/SMAD pathway stimulation induces epithelial-to-mesenchymal transition (EMT) in HepaRG cells. This is evident from the upregulation of EMT-associated genes such as *SNAIL1, SNAIL2, VIM, N-CDH2, ZEB1*, and *ZEB2*. TGF-β also reduces the metabolic activity of CYP enzymes and lowers albumin secretion, highlighting its impact on cellular functions (Zahmatkesh et al., 2022). Advanced systems like the co-culture of THP1 (monocyte cell line), HSC-TERT (immortalized HSCs), and HepaRG on scaffold-free platforms further replicate cellular interactions. Methotrexate and thioacetamide exposure increased macrophage and HSC proliferation while activating cellular defense mechanisms, as indicated by elevated mRNA levels of Nrf2 and Keap1. ECM remodeling, characteristic of fibrosis progression, is also observed in co-cultures treated with these compounds. Dose-dependent upregulation of *COL1A1, COL4A1, FN*, and *CD44* correlates with increased type I collagen and MMP-2 secretion, as well as heightened deposition of type I collagen and α-SMA (Prestigiacomo et al., 2017). More recently, a co-culture system composed of HSC-derived LX-2 cells, primary macrophages and HepaRG cells showed typical features of MASLD (steatosis, fibrogenesis, inflammation) after exposition to a mixture of fatty acids and was proposed as a valuable *in vitro* model for the early detection of at-risk drugs in MASLD patients (Bronsard et al., 2024).

### 4.4 Hepatic stellate cells from iPSCs

Induced pluripotent stem cells (iPSCs) represent a breakthrough in developing cell models for studying diseases, thanks to their ability to self-renew, differentiate into any cell type, and generate models with disease-specific mutations. Several protocols have been established to derive HSCs from iPSCs, mimicking the embryonic development of these cells. The resulting HSCs exhibit a morphology comparable to primary HSCs and display hallmark features of the quiescent state, such as vitamin A storage in lipid droplets, as well as the expression of genes like *DES, PDGFRβ, ALCAM, LRAT, ACTA2, PPARγ*, and *COL1A1*, and the production of proteins including PCDH7, vimentin, type I collagen, fibronectin, and α-SMA (Koui et al., 2021; Lai et al., 2022; Martinez Garcia de la Torre et al., 2025; Vallverdu et al., 2021).

Like primary HSCs and established cell lines, iPSC-derived HSCs have proven to be effective as *in vitro* models for fibrosis. When exposed to profibrotic stimuli, such as PDGFRβ, these cells become activated, showing increased proliferation and migration. Moreover, treatment with TGF-β further enhances the expression of profibrotic genes such as *COL1A1, COL3A1, ACTA2, TIMP1*, and *TGFB*, as well as the production of type I collagen, nestin, and α-SMA (Coll et al., 2018; Lai et al., 2022). Recently, the Retinoic Acid Related Oprhan Receptor Alpha has been identified as a key transcription factor which is needed for HSC differentiation, commitment and activation (Martinez Garcia de la Torre et al., 2025).

In a Transwell system, exposure to APAP and thioacetamide induced the expression of *COL1A1, COL3A1*, and *ACTA2* in HSCs derived from iPSC. Moreover, these models have also been validated for studying virus-induced fibrosis, as exposure to hepatitis B or C viruses enhances the expression of *COL1A1*, *COL3A1*, and *ACTA2*, along with increased production of type I collagen and α-SMA in iPSC-derived HSCs cocultured with HepG2 and Huh7.5 cells, respectively (Lai et al., 2022).

On the other hand, iPSC-derived HSCs co-cultured with HepaRG cells in spheroids allow for the evaluation of profibrotic molecule deposition and cellular organization in response to profibrotic stimuli. TGF-β treatment induced the expression of fibrogenesis markers, along with the secretion of pro-collagen type I and increased staining of phalloidin and collagen. Additionally, this system has been used to model drug-induced fibrosis: exposure to APAP activated iPSC-derived HSCs, as evidenced by increased expression of fibrogenesis-related genes and the secretion of pro-collagen type I and α-SMA (Coll et al., 2018).

### 4.5 Organoids

Organoids are 3D structures derived from stem cells that mimic the architecture and basic functions of organs on a smaller scale. These models have revolutionized biomedical research by providing a more accurate representation of human tissue physiology and pathology compared to traditional 2D systems.

Liver organoids are a promising model for studying the development, progression, and treatment of conditions such as fibrosis, steatosis, and liver cancer. They preserve key liver tissue features, including cellular organization, metabolic function, and toxin responses, enabling detailed pathological analyses and offering an ideal platform for personalized drug testing (Nuciforo and Heim, 2021).

In other approximation Wu et al. (2023) generated organoids by differentiating iPSCs into spheroids embedded in a Matrigel matrix enriched with EGF, FGF2, VEGF, CHIR9902, A83-01, ascorbic acid, and retinoic acid to promote the specification of parenchymal and non-parenchymal cells. After 20 days, the organoids expressed markers of hepatocytes, HSCs, macrophages, and cholangiocytes. Methotrexate treatment for 7 days in these spheroids increased type I collagen deposition and lipid accumulation, hallmarks of fibrosis and steatosis. Profibrotic markers such as *COL1A1, ACTA2, SMAD7, TGFB1*, and *TIMP1* were also upregulated (Wu et al., 2023).

Finally, organoids are valuable tools for studying congenital hepatic fibrosis. Autosomal recessive polycystic kidney disease (ARPKD), a monogenic disorder affecting the kidney and liver, leads to progressive hepatic fibrosis, the primary cause of mortality in surviving patients. Organoids derived from iPSCs with PKDHD1 mutations replicate the hepatic disease phenotype. These organoids show irregular bile duct formation and ECM deposits occupying 25%–30% of their volume. Cholangiocytes are immature, with activated TGF-β signaling driving collagen fiber formation. Increased myofibroblast generation, enhanced STAT3 pathway activity, collagen production, and PDGFRB expression further contribute to the pathogenesis of hepatic fibrosis (Guan et al., 2021).

### 4.6 Precision-cut liver slices

Cell cultures derived from individual cells fail to fully replicate the complex cellular interactions that occur *in vivo* in the liver. Liver slices have emerged as valuable tools for studying liver diseases because they can preserve the cellular interactions present in the original organ (Fisher and Vickers, 2013).

The use of liver slices offers the possibility not only to create fibrosis models by exposing healthy donor cultures to profibrotic molecules but also to generate fibrosis models using liver slices from patients with liver fibrosis at different stages. Liver slices from patients at various fibrosis stages display fibrotic markers such as α-SMA and collagen type 1 (de Mesquita et al., 2017).

Moreover, liver slices respond to various fibrosis inducers, including viruses, alcohol, and fat. For instance, exposing liver slices from healthy donors or fibrosis patients to ethanol, HCV, or palmitate leads to an increased expression of fibrosis markers such as TGF-β1, α-SMA, HSP47, MMP-2, MMP-9, ProCol1A1, and VEGF. This increase is more pronounced in liver slices obtained from fibrosis patients. Additionally, a synergistic effect between different fibrosis inducers has been observed, accelerating fibrosis progression (Kartasheva-Ebertz et al., 2021). Despite the results obtained using liver slices, they still present important limitations such as poor availability of fresh human liver tissue or significant technical requirements, that limit their wider application.

## 5 Overcoming 2D model limitations with new 3D model approaches

Liver fibrosis is a complex and multifaceted pathological process characterized by different mechanisms involving the interactions among various cell types, signaling pathways, and biomechanical changes, posing challenges for researchers in understanding the disease and developing effective therapies (Mazza et al., 2017). 3D models offer significant advantages over 2D models in replicating the complex microenvironment of the liver. While 2D models have been valuable for their simplicity, reproducibility and ease of use, they fail to mimic the intricate spatial interactions and microenvironmental cues critical in liver pathophysiology. In contrast, 3D models effectively recreate essential aspects of the liver microenvironment, including cell-cell and cell-ECM interactions, signaling pathways, cellular migration, chemotaxis, gradients of oxygen and growth factors. These features enable 3D models to support cellular phenotype more effectively, resulting in a more accurate representation of liver fibrosis for research and drug testing purposes (van Grunsven, 2017). Although 3D *in vitro* models have shown increased functionality, within the setting of liver disease models, controlling the properties of the ECM is basic to understand the start of the disease as well as its progression and regression (Carvalho et al., 2024). In this sense, the use of biomaterials that allow to understand how biomechanical and biochemical network signals impact cell behavior would provide a deeper understanding of liver fibrosis. In fact, myofibroblasts’ differentiation of HSCs is encouraged by a stiffer matrix, which also increases the release of ECM. Additionally, stiffness enhances the expression of MMPs’ inhibitors while downregulating the expression of MMPs (Lachowski et al., 2019). In the last years, several 3D *in vitro* models have been developed to recreate the complex microenvironment of the fibrotic liver: hydrogels, scaffolds and liver-on-chips (Gong et al., 2023). Biomaterials can be generally classified according to their origin as natural or synthetic. Natural biomaterials such as collagen and hyaluronic acid have been widely used because of their biocompatibility and interaction with cells, although their mechanical properties are commonly weak and hard to fine-tune. On the contrary, synthetic materials such as polyacrylamide provide tunable and reproducible mechanical properties, but do not interact with cells (Perez et al., 2013). Table 2 exemplifies 3D cellular systems used for modelling liver fibrosis.

**TABLE 2:** Recent 3D *in vitro* model for liver fibrosis.

3D model	Material and crosslinking	Cells	Fibrosis induction or inhibition	Highlights	References
Hydrogel	MeHA	Primary HSCs	Mechanical induction: increased *in situ* stiffness	- Hepatic stellate cells spread and acquire myofibroblast morphology in response to matrix stiffening - Yap is translocated to the nucleus in stiffer matrices - Crosstalk between YAP and α-SMA: contractility is critical for YAP/TAZ and myofibroblast activation	Caliari et al. (2016a)
	PEG	Progenitor cells	Mechanical induction with stiffness	- Spheroids in higher stiffness exhibited reduced hepatic markers - Increase metalloproteases in high stiffness hydrogels	Sorrentino et al. (2020)
	Collagen Type I	Primary HSCs, LX-2	TGF-β1	- HSCs created a stiffer environment than LX-2 and expressed higher levels of TIMP1 and LOXL2 - The model responds to inhibitors such as Alk5i and the fibrotic phenotype is reduced	Brovold et al. (2020)
	Collagen Type I	HepaRG, primary HSCs, LX-2	TGF-β1	- Activated HSCs induced more severe fibrotic state (higher ECM contraction and rigidity) compared to immortalized LX-2 - Higher expression of fibrosis-related genes (TGF-β, TIMP-1, LOXL2, COL1A2) was noted in the fibrotic environment - Enhanced Notch signaling in activated HSCs presence with higher expansion of CK19+ cells and larger biliary-like cell clusters	Brovold et al. (2021)
	Gelatin crosslinked with transglutaminase	HepG2	Mechanical induction: increased *In situ* stiffness	- The increase of the viscoelasticity with transglutaminase (increased crosslinking) mimicked fibrosis progression in stiffness - Increased viscoelasticity over time resulted in decreased cell viability	Cacopardo and Ahluwalia, (2021)
	Collagen type I	iPSCs-derived HSCs	-	- Maintenance of a population of quiescent iPSCs-derived HSCs in a lowly activated state for up to 5 days - Cells show an inactivated profile due to a soft substrate - Changes in matrix components do not alter the quiescent state	Gong et al. (2023)
	GelMa	HepaRG, LX-2, THP1	TGF-β1 and Methotrexate	- Enhanced activation of LX-2 cells in the THP1 presence - Increased *TIMP1* and *MCP1* in the triple co-culture - Increased IL-6 and IL-1 production in the 3D co-culture - Methotrexate treatment increased *MMP1* expression and decreased *COL1A1* expression	Jung et al. (2024)
Scaffold	Decellularized ECM	Huh7, HFL	-	- Scaffolds preserve the tissue-specific ECM of fibrotic liver - Hepatic cells in fibrotic liver scaffolds acquired the EMT phenotype: overexpression of *ITGβ1* and *pFAK* - Fibrotic scaffolds promoted higher proliferation ability and greater drug resistance of cells	Miyauchi et al. (2017)
	Decellularized ECM	HepG2, LX-2	TGF-β1	- Cirrhotic scaffolds promote pro-fibrogenic and pro-cancerogenic progression - TGF-β1 increased markers of HSC activation and ECM production - Sorafenib demonstrated anti-fibrotic effects in the co-culture model, reducing pro-fibrogenic markers and STAT3 phosphorylation	Thanapirom et al. (2021)
	Bioprinted GelMA	HepaRG, LX-2, HUVEC	TGF-β1	- Long-term culture (28 days) And improved hepatocyte phenotype and functionality compared to monolayers - Increased expression of fibrogenic genes (*ACTA2, COL1A1*) after TGF-β1exposure - Increased fibrillar collagen deposition	Cuvellier et al. (2021)
	PVA Gel	HepG2	-	- Scaffolds with tunable porosity and tensile strength - Long-term culture (21 days) - Increased albumin production	Vasanthan et al. (2015)
	3D ECM scaffolds from human healthy and cirrhotic livers	HSCs (PNPLA3 I148 M variant)	TGF-β1	- PNPLA3 I148 M HSCs showed mitochondrial dysfunction and damaged antioxidant responses - HSCs carrying PNPLA3 I148 M variant exhibited increased TGFβ1 signalling, that reduced antifibrotic NR4A1 activity - Culture on cirrhotic ECM worsens HSCs’ behavior, demonstrating how the fibrotic microenvironment influences progressive chronic liver diseases	Caon et al. (2024)
	Protein-conjugated PEG microgels	HSCs	-	- Microgel protein content modulates HSC morphology and protein and gene expression (i.e., *ACTA2, MMP2, IL6*) - The composition of the scaffolds modulates ECM remodelling and HSC functionality	Ryoo et al. (2024)
	Col/FN electrospun	PHH + LX-2	Alcohol	- Collagen:Fibronectin_(3:1) scaffolds showed improved intra and intercellular interactions between PHHs and LX-2 - Alcohol treatment produced a decrease in albumin expression and an upregulation of *CYP2C9, CYP3A4,* *CYP2E1* and *CYP1A2*	Das et al. (2021)
	Fibronectin-Polyacrylamide	HSCs	Stiffness modulation	- Scaffolds with 4, 12, and 25 kPa rigidities represented the progression from healthy to fully liver fibrosis - HSCs were cultured on polyacrylamide substrates with increased rigidity showed downregulation of intracellular protein levels of MMP-9 and TIMP-1	Lachowski et al. (2019)
On-a-chip	Collagen membrane sandwiched and two PDMS layers	C3A, HepG2, LSEC	Mechanical stiffness	- Recreation of a liver sinusoid to unravel the co-effect of matrix stiffness and shear flow in hepatic cells - Matrix stiffness and shear stresses regulated hepatocyte functions synergically - Stiffer hydrogel and high shears reduce hepatic functionality	Li et al. (2021)
	Sandwich chip with GelMa hydrogels	HepG2, LX-2, ECs	Mechanical induction (stiffness) and TGF-β1	- Modelling early (8% GelMa) and late (15% GelMa) fibrosis in a Disse space-like structure - Stiffness modulated TGF-β signaling pathway. *SMAD2* and *SMAD3* are upregulated in stiffer substrates (15% GelMa) - Different response to anti-fibrotic drugs was observed in the early model	Liu et al. (2023)

ECM, extracellular matrix; ECs, endothelial cells; GelMa, methacrylate gelatin; HSCs, hepatic stellate cells; ITGb1, integrin b1; MeHA, methacrylate hyaluronic acid; PDMS, polydimethylsiloxane; PEG, polyethylene glycol; PVA, polyvinyl alcohol; TGF-β1, transforming growth factor β1.

Given the intricate nature and diversity of fibrosis development, there are several key functions that researchers can consider in the 3D models. One important clinical marker of fibrosis is the stiffening of the ECM. It significantly affects the activation of HSCs and their transformation into myofibroblasts, making it a focal point in some studies (Liu et al., 2021). In fact, HSCs cultured in conventional plastic plates are activated due to the high tension of the plastic surface (20 GPa), which can lead to artifactual activation of HSCs in these models (Caliari et al., 2016b; Thanapirom et al., 2021). Some models have been designed to prolong the culture of HSCs in the quiescent state. Gong et al. developed a model based on collagen type I hydrogel to generate iPSC-derived HSCs in the quiescent state and maintain them in an inactivated state for up to 5 days. The iPSC-derived HSCs retained their activation capability and were tested for their response to TGF-β1 exposure (Gong et al., 2023). Designing 3D models that exhibit stiffening and biomechanical fibrosis properties (4–5 kPa) is crucial to ensure *in vivo* physiopathology and drug testing (Caliari et al., 2016b; Lemine et al., 2024). Synthetic hydrogels have been selected by many researchers for recreating environments, as they allow for highly controlled properties (Caliari et al., 2016a; Ravichandran et al., 2021; Sorrentino et al., 2020). For example, researchers created polyethylene glycol hydrogels decorated with liver matrix biomolecules (fibronectin, laminin, and collagen IV) to model healthy and fibrotic liver tissues of 1.2 kPa and 4 kPa stiffness, respectively (Sorrentino et al., 2020). They evaluated the behavior of progenitor cells under high stiffness. Cells exhibited reduced liver markers in the fibrotic model with increased MMPs profiles. Other authors prefer using natural biomaterials like collagen, the main component of the liver ECM. For example, Brovold et al. modelled fibrosis in type I collagen hydrogels at different degrees of fibrosis severity (Brovold et al., 2020). They observed that primary HSCs exhibit a higher fibrotic phenotype than the LX-2 line, and both respond to fibrotic inductors and inhibitors. The same model was used to co-culture with undifferentiated HepaRG cells and evaluate the effects on biliary progenitor cells (Brovold et al., 2021). This study proved again that primary HSCs created a more severe fibrotic state than LX-2, characterized by higher ECM contraction and rigidity and increased expression of fibrosis-related genes such as *TGF-β, TIMP-1, LOXL2*, and *COL1A2*. They found a considerable expansion of CK19+ biliary cells, mimicking conditions in congenital biliary diseases closely linked to fibrosis.

Despite the physiological significance of matrix stiffening in fibrosis development, only a few models have successfully recreated it *in situ* (Cacopardo and Ahluwalia, 2021; Caliari et al., 2016b). In their innovative study, Cacopardo and Ahluwalia developed a two-step model with gelatin that effectively mimics liver fibrosis progression. In the first step, cells were encapsulated in the hydrogel. Then, the already-formed hydrogels were exposed to further TG, subsequently increasing gelatin fiber crosslinking, successfully simulating the overproduction of ECM and the associated tissue stiffening. This approach transformed the hydrogels into increasingly elastic and stiffer structures, closely replicating the mechanical changes observed in liver fibrosis (Cacopardo and Ahluwalia, 2021).

Liver-on-a-chip devices are advanced *in vitro* models that combine microfluidics, tissue engineering, and microfabrication. It has been demonstrated that these systems could significantly enhance drug discovery and development, enabling producers to provide safer, more potent medications faster and at a lower cost (Ewart et al., 2022). Organ-on-a-chip replicates liver microarchitecture, metabolic zonation and dynamic blood flow, offering more realistic environments for studying the liver ECM remodeling with the fluid flow (Liu et al., 2023; Mannaerts et al., 2020). Li et al. explored how mechanical factors (stiffness and flow) affect liver cell function in fibrosis using a microfluidic platform with a stiff-tunable collagen membrane (Li et al., 2021). They proved that increased matrix stiffness reduces hepatocyte albumin production and cytochrome P450 reductase expression, while low shear stress enhances hepatocyte function, and high shear stress leads to phenotype loss. This research highlights the complex interplay between matrix stiffness and shear stress in regulating hepatocyte behavior (Li et al., 2021). On the other hand, Lee and cols. developed a 3D liver fibrosis-on-a-chip model using gelatin-based bioinks and cell-printing technology with precise control of cellular delivery (Lee et al., 2020). This study demonstrated the potential to model fibrotic processes with the upregulation of vimentin, α-SMA and desmin and downregulation of CYP3A4 (key markers of HSCs activation). They also observed collagen type I deposition coupled with a reduction in viability verified by the TUNEL assay (Lee et al., 2020). Additionally, liver-on-a-chip devices have been also used for modelling MALSD and MASH, showing the successful recapitulation of the main histologic features and endpoints, emerging as *in vitro* platforms to study disease pathogenesis and to test and develop new treatments (Freag et al., 2021; Kostrzewski et al., 2017).

Current 3D models often fail to fully capture the complex interactions between the liver and the immune system, which are strongly interconnected. Its crosstalk contributes to the development of fibrosis. However, models that are capable of representing this crosstalk are limited. Jung et al. developed 3D models for better understanding the crosstalk between hepatocytes and THP1 cells in methacrylate gelatin (Jung et al., 2024).

Significant progress has been made in developing 3D models for studying liver fibrosis. These 3D models provide a more accurate platform to replicate the biomechanical and biochemical conditions of the hepatic microenvironment, which is crucial for understanding fibrosis progression and evaluating new therapies. However, many mechanisms remain to be understood and modelled, presenting an ongoing challenge in the search for effective treatments. The lack of adequate models that fully capture the complexity of cell-matrix interactions and the biomechanical changes associated with fibrosis limits our ability to discover new therapeutic targets. Therefore, it is essential to continue developing and optimizing these models to advance the treatment of fibrotic liver diseases.

## 6 Assessment of anti-fibrotic treatments *in vitro*

The progression of liver fibrosis is caused by a variety of cells and signaling pathways, which poses a great challenge in clinical therapy. At the moment, the eradication of etiologies is the main focus of liver fibrosis treatment. In the case of hepatic metabolism problems, bariatric surgery and lifestyle modifications have been investigated (Vilar-Gomez et al., 2015). On the other hand, clinical evidence for treating hepatic fibrosis has been generated by antiviral medications for viral hepatitis (D'Ambrosio et al., 2012; Marcellin et al., 2013), which suggests that scarring can be reversed (Mohammed et al., 2023).

Currently, no effective treatment for liver fibrosis is available; thus, as a result, ongoing studies regarding anti-fibrotic therapy are underway. The development of successful anti-fibrotic treatments requires the ability to identify additional mechanisms of liver fibrosis through the understanding of intercellular molecular networks, as well as to target specific cell types (Odagiri et al., 2021). Numerous factors, including the overproduction and secretion of pro-inflammatory cytokines, the rise in hepatocyte apoptosis, the proliferation of activated HSC, and the excessive production and deposition of ECM, will contribute to the imbalance of pro-fibrosis/antifibrosis mechanisms and facilitate the onset and progression of liver fibrosis (Campana and Iredale, 2017; Roehlen et al., 2020). In contrast, a number of factors, such as the increase of anti-inflammatory cytokines, hepatocyte proliferation, apoptosis and the restoration of the resting phenotype of activated HSC, and an increase in the degradation of ECM, successfully prevent the occurrence and progression of liver fibrosis, postpone liver fibrosis, and even reverse the process and go back to the normal functionality and structure (Campana and Iredale, 2017; Roehlen et al., 2020; Tan et al., 2021). Consequently, developing drugs that can balance pro- and anti-fibrotic events is essential for developing effective anti-fibrotic drugs. Table 3 provides examples of anti-hepatic fibrotic drugs assessed in clinical trials, highlighting their mechanisms of action.

**TABLE 3:** Examples of therapeutic strategies for liver fibrosis and target cells or pathways.

Drug/Therapeutics	Modality	Phase/NTC	Target	Mechanism of action	References
Obeticholic acid	Small molecule	III/NCT02548351	FXR agonist	- Decreased HSC activation - Suppression of metabolic stress-induced p53 activation and cell death in hepatocytes	Goto et al. (2018); Verbeke et al. (2016)
Resmeritom	Small molecule	III/NTC03900429	THR-β agonist	- Increased lipophagy, mitochondrial biogenesis and mitophagy, stimulating increased hepatic fatty acid β-oxidation	Karim and Bansal, (2023)
Aramchol	Small molecule	III/NTC02279524	SCD1 inhibitor	- Inhibition of *de novo* lipogenesis in hepatocytes - Inhibition of transdifferentiation and activation of HSC	Ratziu et al. (2021)
Lanifibranor	Small molecule	III/NCT04849728	PPAR inhibitor	- HSC deactivation - Improvement of hepatocyte’s functionality - LSEC deactivation - Downregulation of cytokines expression in macrophages	Boyer-Diaz et al. (2021)
Pegbelfermin	Biomacromolecule	II/NTC03486912	FGF21 analogue	- Inhibition of gluconeogenesis in hepatocytes	Sanyal et al. (2019)
Cenicriviroc	Small molecule	III/NTC03028740	CCR2 and CCR5 inhibitor	- Reduced monocyte/macrophage recruitment - M2 macrophage polarization	Neokosmidis and Tziomalos, (2018)
Emricasan	Small molecule	II/NTC02138253	Pan-caspase inhibitor	- Inhibition of hepatocyte apoptosis - Reduction of inflammation and fibrosis	Barreyro et al. (2015)
Hydronidone	Small molecule	II/NTC02499562	HSC proliferation inhibitor	- Inhibition of HSC activation - Upregulation of Smad7 in HSC cells - Smad7-mediated TGFβRI degradation and inhibition of the TGF-β signaling pathway	Liu et al. (2017)
Liraglutide	Small molecule	II/NCT01237119	GLP-1 receptor agonist	- Reduction of the activation of KC and HSC - Effects on carbohydrate metabolism	Perakakis et al. (2021)
Simtuzumab	Antibody	II/NTC01672866	Inhibition of LOXL2	- Decrease of the stability of ECM by antagonizing collagen cross-linking	Meissner et al. (2016)

ECM, extracellular matrix; HSC, hepatic stellate cell; KC, kupffer cell; MASH, metabolic dysfunction-associated steatohepatitis; TGF-β1, Transforming Growth Factor β1.

Antifibrotic drug candidates can be tested early in the drug development process using different preclinical models. The well-known interspecies differences in drug metabolism, pharmacokinetics and drug targets limit the prediction of drug effects using animal models. Human cell models offer advantageous tools for assessing the anti-fibrotic potential of drugs as well as allowing studying the potential mechanisms implicated and even deciphering the cell targets implicated in drug responses. Due to the multicellularity and complexity of the fibrotic process, in recent years, multicellular models have been widely used for the efficacy assessment of these drugs. For inducing the profibrotic phenotype, different stimuli can be used, although the use of TGF-β1 is one of the most common factors used in *vitro* assessments. Table 4 summarizes some of the cell models used and the major outcomes of these models for assessing anti-fibrotic therapies.

**TABLE 4:** Examples of preclinical *in vitro* models for the assessment of anti-hepatic fibrosis molecules.

*In vitro* model	Induction of fibrosis	Anti-fibrotic drugs	Outcomes/Comments	References
Human liver myofibroblasts	TGF-β	Pirfenidone Losartan (negative control)	- α-SMA expression was chosen as a reliable activation marker - Coll1 was previously chosen as a marker for ECM production - Human liver myofibroblasts could reflect inter-patient variability in fibrosis progression	Aoudjehane et al. (2016)
Human precision-cut liver slices, HSC and LX-2 cells	7-day plastic activation	Liraglutide	- Amelioration in HSC phenotype when treated with liraglutide	de Mesquita et al. (2017)
PHH, human skin stem cell-derived hepatic cells (hSKP-HPC), HepaRG, HepG2 and LX-2 cells	FA and inflammatory cytokines (TNF-α, IL-1β, and TGF-β)	PPAR agonists (bezafibrate, elafibranor, fenofibrate, lanifibranor, pemafibrate, pioglitazone, rosiglitazone, and saroglitazar)	- The comparative study in different cell models showed that PHH, hSKP or a combination of both as the most sensitive models for determining anti-NASH responses - PPAR agonists produced differential responses regarding the reduced expression of profibrotic genes, inflammatory chemokine production, and fat accumulation, allowing creating a score system to grade potencies - Elafibranor and saroglitazar showed the strongest anti-NASH properties	Boeckmans et al. (2021)
PHH + NPC spheroids	Spontaneous, FFA or TGF-β	Cenicriviroc, Elafibranor, lanifibranor	- A fibrotic phenotype (expression of COL1A1 and SMA) was found in a number of spheroids, either spontaneously (mostly in PNPLA3 mutant donors) or in response to FFA. - The incubation with anti-fibrotic compounds for 7 days prevented fibrillary deposition	Hurrell et al. (2020)
Human liver slices	TGF-β Alcohol Virus infection	A-tocopherol Ursodeoxycholic	- The model remains viable up to 21 days, allowing long-term studies - The described test system reproduces liver fibrogenesis related to HCV infection, ethanol or FA exposure - A significant decrease in TGF-β and procollagen1A1 expression and in triglycerides production after anti-fibrotic therapy	Kartasheva-Ebertz et al. (2021)
LX-2 + HepG2 in 3D healthy and cirrhotic human liver scaffolds	TGF-β	Sorafenib	- Gene expression and pro-collagen1 secretion were 1–3 times more abundant in cells cultivated in 3D cirrhotic compared to healthy scaffolds - Co-cultures 3D ECM scaffolds can reproduce molecular and cellular processes that result from TGF-β exposure and cause fibrosis - The pro-fibrogenic effects of TGF-β1 were markedly inhibited by sorafenib, and a downregulation of STAT3 phosphorylation	Thanapirom et al. (2021)
Human liver organoids	TGF-β Methotrexate LPS	60 componds (i.e., SD208, Imatinib, Cilofexor, Silymarin)	- It modelled liver fibrogenesis after incubation with TGFβ or LPS. - Collagen I staining was employed as a readout for liver fibrosis using high-content analysis technology - Gene expression analysis showed that SD208 and Imatinib downregulated the expression of different fibrogenic (i.e., *ACAT2, COL1α1, LOX, TIMP1)*	Wu et al. (2023)
Liver microtissue (Huh-7, THP-1, and LX-2 and HUVEC cells)	OA + PA	Liraglutide Withdrawal of FFA	- Steatotic profile of liver tissues was established after 3 days and advanced to initial fibrosis by day 8 - Liraglutide significantly reduced MALSD profile at different stages, including fibrotic markers (i.e., *COL1A1* expression)	Asadollahi et al. (2024)
Human liver spheroids (hepatocytes, HSCs, and NPCs)	MASH cocktail (OA, PA, Fructose, glucose, LPS, TGF-β)	Supression of metabolic injury	- Human liver spheroids incubated with MASH cocktail upregulate lipid accumulation, expression of FFA-regulating enzymes *ACSL4* and *CPT1A*, and raise mRNA levels of inflammatory cytokines such as *CXCL1, IL6 and IL8* - After stopping the metabolic injury, the expression of fibrogenic genes was decreased	Kim et al. (2024)
HSC derived from iPSC	Activation by plastic dish culture for 7 days	Lanifibranor, SB431542, Dorsomorphin, retinoic acid, palmitic acid and Y27632 + screening	- Actin score (quantification of F-Actin accumulation) is defined as an indicator activated HSC that can be used as a screening tool - The combination of test compounds, reduced the expression - of activation marker genes (*ACTA2*, *COL1A1*) and increased the expression of *LHX2* and *LRAT* which are expressed in quiescent cells	Nakano et al. (2024)
PHH, NPC and LSECs spheroids	FFA	Lanifibranor, mevastatin, cencrivroc, molsidomine, SB-525334, BI1467335, AGI-1067	- TIMP1 knockdown in the test system led to a reduction in *COL1A1* accumulation as well as a decreased pro-COL1A1 in the medium - Differential responses to anti-MASH drugs were observed in spheroids with and without LSEC. For instance, AGI-1067 (a VCAM-1 inhibitor) decreased *COL1A1* only when LSEC were present	van Riet et al. (2024)

3D, three-dimensional; αSMA, α-smooth muscle actin; FFA, free fatty acids; HSC, hepatic stellate cells; iPSC, induced pluripotent stem cell; KC, kupffer cell; LPS, lipopolysaccharide; LSECs, liver sinusoidal endothelial cells; NPC, non-parenchymal cells; OA, oleic acid; PA, palmitic acid; PHH, primary human hepatocyte; TGF-β1, transforming growth factor β1.

It should be considered that liver fibrosis is associated with changes in ECM components and mechanical properties that can promote the progression of the disease in more serious stages. Therefore, by controlling ECM characteristics and improving the understanding of disease mechanisms, the regression of liver fibrosis could be achieved *in vitro* and finally translate the results to the clinical practice (Carvalho et al., 2024). In this sense, there are still many challenges of bridging preclinical findings to clinical trials since standardization and validation are key factors to contemplate (Carvalho et al., 2024). The “Guidance Document on Good *In Vitro* Method Practices” published by the Organisation for Economic Co-operation and Development (OECD) describes how to standardize procedures for guaranteeing their appropriate design, description and reliability for their use in a regulatory context (OECD, 2018). This guideline could be used to improve the consistency and applicability of new *in vitro* platforms. On the other hand, validation is defined as the process by which the reliability and relevance of a procedure are established for a specific purpose (OECD, 2005). So, following adequate method development and parameter definition, an internal validation procedure should be carried out to evaluate the *in vitro* method’s repeatability, selectivity, sensitivity, and stability. Validation guarantees a methodical and scientific assessment of *in vitro* techniques and approaches and sits at the nexus of *in vitro* method development and optimization, regulatory approval, and translational medicine.

## 7 Conclusions and future perspectives

For an in-depth examination of the processes underlying liver fibrosis, cell-based models are essential. The limited primary cell supply and lack of heterotypic essential cell–cell interactions are two of the drawbacks of the monoculture of HSCs. In this sense, co-cultures might be superior *in vitro* models since they enable the interactions between HSCs and other hepatic cells (i.e., hepatocytes, KCs), which are essential to the start of the fibrotic process. On the other hand, stem cell-based systems, which are based on the differentiation of stem cells of various origins into mature HSCs, represent a very promising test system that would allow to obtain different hepatic cells for studying liver disease (Asahina et al., 2009; Miyata et al., 2008). Additionally, using 3D models allows to mimic cell-cell and cell-ECM interactions as well as nutrients and oxygen gradients in order to better replicate liver microenvironment. Optimized multicellular 3D cultures will provide robust and accurate *in vitro* systems for disease modelling. These platforms will improve translational research to increase knowledge of mechanisms and factors involved in disease progression and help to identify potential new biomarkers for early diagnosis of liver fibrosis. However, current models do not completely recapitulate liver’s structure since vascularization should be also considered. Moreover, other immune cells different from KC would also help to better mimic the immune-mediated pathomechanisms in MASLD and liver fibrosis (Bronsard et al., 2024). It is clear that multicellular interaction within a pro-fibrotic microenvironment leads to liver fibrosis, thus a key issue to achieve the reversal of liver fibrosis may be the restoration of microenvironmental homeostasis, which could help all types of liver cells to maintain a more stable and long-lasting state and prevent liver cells from changing into a profibrotic state (Meng et al., 2022). In this sense, the identification of reliable biomarkers of fibrosis *in vitro* would help to clearly make a step forward in the translation to the clinical setting.

In addition, such *in vitro* platforms will contribute to high-throughput assessment of new anti-fibrotic drugs with potential to revert the disease. Thus, these new methods and technologies will surely aid in the creation of effective clinical treatment plans for liver fibrosis, which will improve human health.

## Abbreviations

2D, two-dimensional; 3D, three-dimensional; αSMA, α-smooth muscle actin; ADAM, A disintegrin and metalloproteinases; APAP, acetaminophen; ARPKD, autosomal recessive polycystic kidney disease; DAMPs, damage-associated molecular patterns; ECM, extracellular matrix; EMT, epithelial-to-mesenchymal transition; HSC, hepatic stellate cells; HUVEC, human umbilical vein endothelial cells; IL, interleukin; iPSC, induced pluripotent stem cells; KC, Kupffer cell; LOX, lysyl oxidase; LSECs, liver sinusoidal endothelial cells; MASH, metabolic dysfunction-associated steatohepatitis; MASLD; metabolic-dysfunction associated liver disease; MMPs, metalloproteinases; NPC, non-parenchymal cells; OECD, Organisation for Economic Co-operation and Development; PDGF, platelet-derived growth factor; PHH, primary human hepatocytes; ROS, reactive oxygen species; TG, transglutaminases; TGF-β, transforming growth factor-β; TIMPs, tissue inhibitors of MMPs; TNFα, tumor necrosis factor α.
